# Acidobacteria Subgroups and Their Metabolic Potential for Carbon Degradation in Sugarcane Soil Amended With Vinasse and Nitrogen Fertilizers

**DOI:** 10.3389/fmicb.2019.01680

**Published:** 2019-07-30

**Authors:** Miriam Gonçalves de Chaves, Genivaldo Gueiros Z. Silva, Raffaella Rossetto, Robert Alan Edwards, Siu Mui Tsai, Acacio Aparecido Navarrete

**Affiliations:** ^1^Cell and Molecular Biology Laboratory, Center for Nuclear Energy in Agriculture, University of São Paulo, Piracicaba, Brazil; ^2^Computational Science Research Center, San Diego State University, San Diego, CA, United States; ^3^São Paulo′s Agency for Agribusiness Technology APTA-SAA, Piracicaba, Brazil; ^4^Department of Environmental Sciences, Federal University of São Carlos, Sorocaba, Brazil

**Keywords:** soil metagenome, DNA microarray, mineral and organic fertilizers, carbon cycling, microbe-mediated process in soil

## Abstract

Acidobacteria is a predominant bacterial phylum in tropical agricultural soils, including sugarcane cultivated soils. The increased need for fertilizers due to the expansion of sugarcane production is a threat to the ability of the soil to maintain its potential for self-regulation in the long term, in witch carbon degradation has essential role. In this study, a culture-independent approach based on high-throughput DNA sequencing and microarray technology was used to perform taxonomic and functional profiling of the Acidobacteria community in a tropical soil under sugarcane (*Saccharum* spp.) that was supplemented with nitrogen (N) combined with vinasse. These analyses were conducted to identify the subgroup-level responses to chemical changes and the carbon (C) degradation potential of the different Acidobacteria subgroups. Eighteen Acidobacteria subgroups from a total of 26 phylogenetically distinct subgroups were detected based on high-throughput DNA sequencing, and 16 gene families associated with C degradation were quantified using Acidobacteria-derived DNA microarray probes. The subgroups Gp13 and Gp18 presented the most positive correlations with the gene families associated with C degradation, especially those involved in hemicellulose degradation. However, both subgroups presented low abundance in the treatment containing vinasse. In turn, the Gp4 subgroup was the most abundant in the treatment that received vinasse, but did not present positive correlations with the gene families for C degradation analyzed in this study. The metabolic potential for C degradation of the different Acidobacteria subgroups in sugarcane soil amended with N and vinasse can be driven in part through the increase in soil nutrient availability, especially calcium (Ca), magnesium (Mg), potassium (K), aluminum (Al), boron (B) and zinc (Zn). This soil management practice reduces the abundance of Acidobacteria subgroups, including those potentially involved with C degradation in this agricultural soil.

## Introduction

Acidobacteria are among the most widespread bacterial phyla that occur in soils around the world, including the tropical soils under sugarcane *Saccharum* spp. (Rachid et al., 2013; Navarrete et al., 2015a; Val-Moraes et al., 2016). The presence of membrane transporters and the use of carbon (C) sources ranging from simple sugars to more complex substrates, such as hemicellulose, cellulose and chitin, are among the genomic and physiological characteristics that may contribute to the survival and growth of Acidobacteria in soil (Ward et al., 2009; Rawat et al., 2012). Kielak et al. (2016) recently reviewed the genomic and physiological characteristics of Acidobacteria and showed that there are still many gaps to understanding the functional role of this bacterial phylum in the soil C degradation process. Despite of this lack of biological and ecological information for Acidobacteria, previous studies in agricultural soils have shown that both microbial C degradation processes and acidobacterial community can be affected by soil management (Craine et al., 2007; Navarrete et al., 2015a; Omori et al., 2016; Wang et al., 2018; Lian et al., 2019).

The soil management practices used in sugarcane cultivation require synthetic mineral fertilizers nitrogen/phosphorus/potassium-NPK (Heffer and Prud’homme, 2008), micronutrients and complete recycling of byproducts of the ethanol and sugar production in sugarcane production fields in the form of organic fertilizer (Mutton et al., 2010). Vinasse is a byproduct of the ethanol industry produced at a ratio of ten to eighteen liters for each liter of ethanol produced (Gasparotto et al., 2014). The chemical composition of vinasse varies with the ethanol plant in which it was generated and the distillation process, although it generally consists of water (93%) and organic and mineral compounds (7%) (Christofoletti et al., 2013). Vinasse has high levels of organic matter but low concentrations of N (0.97 to 4.75 g L^-1^) and P (1 to 190 mg L^-1^) and high C:N ratio (Moran-Salazar et al., 2016). The main non-aqueous component of vinasse is organic matter in the form of glycerol, organic acids and yeast (Christofoletti et al., 2013). Depending on the most used in the sugarmill fermentation process, vinasse has also high concentration of potassium (K), calcium (Ca) and sulfur (S), medium concentration of magnesium (Mg) and micronutrients (Christofoletti et al., 2013). Since the 1960s, vinasse has been used as a fertilizer in sugarcane production fields to solve the ecological problem of its disposal in water sources like rivers and lagoons. Studies from the 1980s have recommended the use of N fertilizer in combination with vinasse in sugarcane fields with high productivity.

The addition of vinasse in soils under sugarcane causes changes in the soil microbial community and chemical processes (Montenegro Gómez et al., 2009; de Barros et al., 2010; Jiang et al., 2012), including organic matter decomposition (Resende et al., 2006). Omori et al. (2016) reported increased bacterial diversity after the application of vinasse to soil under sugarcane and showed that the Acidobacteria subgroups Gp3 and Gp4 were more abundant in soil fertilized with vinasse. In turn, Navarrete et al. (2015a) showed that the Acidobacteria subgroups Gp4, Gp11, Gp17, Gp21, and Gp25 were positively related to chemical factors of the soil fertilized with N and vinasse compared with soils fertilized with N alone and soils without N fertilizer and vinasse. Soils fertilized with N and vinasse usually present high levels of sulfur S, K and total C and increased pH (Glória and Filho, 1983). The addition of N as fertilizer may decrease recalcitrant C decomposition (Craine et al., 2007; Wang et al., 2018), which may affect members of the bacterial community that act as decomposers and play a vital role in the C cycle.

Because of the substantial effects that soil agricultural management has on carbon-degrading microorganisms in soils, we would like to obtain better insight into the Acidobacteria community in sugarcane cultivated soil. For this purpose, the present study was designed to evaluate the response of Acidobacteria subgroups to the addition of N and vinasse in tropical soil under sugarcane and the metabolic potential of the subgroups in the degradation of C in these soils. For this purpose, soil genomic DNA shotgun sequencing was performed to identify the Acidobacteria subgroups. A high-throughput functional gene array termed “GeoChip v. 5.0M” was used for the large-scale quantification of the functional microbial genes associated with C degradation. While soil genomic DNA shotgun sequencing-based approach is useful to detection of Acidobacteria subgroups based on taxonomic identification of soil metagenome sequences (Navarrete et al., 2015a), high-throughput functional gene array is a powerful and high-performance tool to analyze specific genes associated with microbe-mediated processes in different habitats (Zhou et al., 2008). Soil chemical analyses were performed, and the results were correlated with the abundance of Acidobacteria subgroups and functional genes associated with soil C degradation using statistical and computational methods.

## Materials and Methods

### Mesocosm Experiment and Soil Sampling

A greenhouse experiment with sugarcane *Saccharum* spp. variety CTC-02 was performed over 150 days between April and December 2013 to normalize certain environmental parameters, such as the moisture regime and soil type. This sugarcane variety has medium to late maturity and presents high productivity and longevity, and the seedlings used in this study were obtained by *in vitro* fertilization and tissue culture. The soil used in the experiment was a clayey-loamy dark red podzolic soil according to the Brazilian Soil Classification collected in the 0-20 cm layer of the Areião Farm, which belongs to the Escola Superior de Agricultura Luiz de Queiroz, Universidade de São Paulo ESALQ-USP in Piracicaba, São Paulo, Brazil 22 42′ 30″ S; 47 38′ 00″ W. On a 15 cm layer of washed crushed stones, 90 kg of soil was added to each of the nine mesocosms 100 L pots (Navarrete et al., 2015a), which received mineral fertilization at planting consisting of 150 kg ha^-1^ of P_2_O_5_ triple superphosphate and 80 kg ha^-1^ of KCl potassium chloride. These mineral fertilizers were incorporated within the soil using a paddle mixer. Three treatments with three replicates each were established according to the N fertilizer source: N0 Control, no N fertilizer; N60, N fertilizer in the form of urea; and NV, urea supplemented with vinasse. Urea (450 g of N kg^-1^) was added in the 5-10 cm soil layer at a dose of 60 kg ha^-1^, and it was immediately mixed into the soil to prevent volatilization. Vinasse was used to irrigate the soil surface at a dose of 0.06 L kg^-1^ (120 m^3^ ha^-1^) as a source of K in addition to organic matter and other nutrients. The treatments that did not receive V received an equivalent volume of water. The KCl dosage was calculated minus the equivalent input of K in case of V treatment according to previous measurements of K content in vinasse samples. The soil moisture content in the pots was maintained at 80% of field capacity throughout the experiment using a moisture sensor (Extech MO750, Nashua, NH, United States). Initially, ten sugarcane seedlings were placed in each mesocosm in order to ensure a rapid influence of the plants on the soil microbiota, and they were periodically removed in pairs to keep the root system below the pot capacity limit, with bulk soil and rhizosphere detachable.

For each mesocosm, three bulk soil samples (about 100 g each sample) were collected from topsoil layer (0 to 10 cm) at equidistant positions within an equilateral triangle (ray equivalent to 1/3 of circular surface area of the pot) using sterile PVC tubes (15 cm in length and 5 cm in diameter) for both chemical and molecular analyses. These samples were collected on the 7th and 150th days after fertilizer application based on the maximum and minimum carbon dioxide (CO_2_) and nitrous oxide (N_2_O) emissions from soil (Navarrete et al., 2015a). The samples were immediately processed after collection for the chemical analyses. For molecular analyses, one subsample was taken from each of the three bulk soil samples for each mesocosm after undeform each one in a plastic bag separately, and they were transported to the Laboratory of Cellular and Molecular Biology of Centro de Energia Nuclear na Agricultura CENA-USP, stored at -20°C and processed within 72 h.

### Analysis of Soil Chemical Factors

The soil samples were air dried at room temperature and sieved through a 0.149-mm sieve to determine the total C and N by dry combustion using a LECO CN 2000 elemental analyzer (PerkinElmer, Waltham, MA, United States). The soil fertility factors analyzed were as follows: pH, potential acidity hydrogen (H + aluminum (Al)), Ca, K, Mg, P, S, micronutrients - iron (Fe), manganese (Mn), zinc (Zn), copper (Cu) and boron (B), exchangeable bases, cation exchange capacity (CEC) and base saturation (BS).

The chemical factors of each soil sample were determined according to Empresa Brasileira de Agropecuária-Embrapa [EMBRAPA] (2011). Soil pH was determined in a soil/0.01 M CaCl_2_ 1:5 suspension. Boron was obtained by hot water extraction. Al, Ca, and Mg were extracted with 1 M potassium chloride. Ca and Mg were determined by spectrometric atomic absorption, whereas Al was determined by acid-base titration. Available P and K were extracted by ion-exchange resin and determined by colorimetry and atomic emission spectroscopy, respectively. The combined results were used to calculate the exchangeable bases sum of Ca, Mg, and K, CEC sum of Ca, Mg, K, Al and H, BS, percentage ratio between BS and CEC and potential acidity H + Al using an equation based on the Shoemaker-McLean-Pratt SMP pH-buffer method.

### Extraction and Sequencing of Soil Genomic DNA

Genomic DNA was extracted from 250 mg of soil obtained from each subsample (avoiding small pieces of roots) using the PowerLyzer^®^PowerSoil^®^DNA Isolation kit (MoBio Laboratories, Carlsbad, CA, United States) following the manufacturer’s instructions. The DNA isolated from each of the three soil subsamples per mesocosm was then pooled and concentrated using the Genomic DNA Clean and Concentrator kit (Zymo Research Corporation, Irvine, CA, United States), constituting a single DNA sample per mesocosm. The concentrated DNA was resuspended in 20 μL of PCR water, and both purity and quality of the genomic DNA were assessed via spectrophotometry on a NanoDrop apparatus (NanoDrop^®^ND-1000 NanoDrop Technologies, Inc., Wilmington, DE, United States) to determine the absorbance at the following wavelengths: 230, 260, 280, and 320 nm. The DNA concentration was determined with the Quant-iT PicoGreen kit (Molecular Probes/Invitrogen, Carlsbad, CA, United States). DNA was stored at -20°C until use.

Eighteen DNA sequencing libraries were prepared using the Illumina Nextera sample preparation kit (Illumina, San Diego, CA, United States) according to the manufacturer’s instructions. The libraries were evaluated on 2100 Bioanalyzer using High Sensitivity DNA kit (Agilent, Santa Clara, CA, United States) to estimate the library size. Libraries were quantified using Qubit dsDNA HS kit on a Qubit 2.0 fluorometer (Life technologies, Carlsbad, CA, United States) and KAPA SYBR FAST qPCR Master mix and Illumina standards and primer premix (KAPA Biosystems, Wilmington, MA, United States) according to the Illumina suggested protocol. The resulting DNA libraries were denatured with NaOH, diluted to 8 pM in Illumina’s HT1 buffer, and spiked with 1% PhiX. Equal concentration of libraries was loaded on MiSeq Reagent v2 sequencing reagent kit (Illumina, San Diego, CA, United States). The equipment used for shotgun metagenomic sequencing was a MiSeq Personal Sequencing System by Illumina (Illumina, San Diego, CA, United States) operated in Rapid Run Mode to generate 2 × 250 pb paired-end reads. In summary, we captured an average of 105.5 MB of genomic sequences per sample.

### Shotgun Metagenomic Data Processing and Taxonomic Identification of Acidobacteria Subgroups

First, paired-end reads were merged using *FLASH* v. 1.2.5 (Magoč and Salzberg, 2011) to produce consensus sequences. Quality control of the consensus sequences was performed using the *Phred* quality score 20 to each base call (Ewing and Green, 1998) with an executable default script in *SeqClean*^1^, and the low-quality bases were removed. Shotgun sequencing of soil DNA from samples resulted in approximately 547.415 thousand merged sequence reads and 375.429 thousand not merged sequence reads after the quality-based filtering procedure. The unmatched trimmed sequences were concatenated in a single file for the metagenomic dataset, which is available on the Metagenomics Rapid Annotation (MG-RAST) server under the “Metagenome of sugarcane soil - CENA/USP” project with accession numbers from 4582104.3 to 4582153.3.

Sequencing data were analyzed using MG-RAST software version 3.2^2^ (Meyer et al., 2008) with default settings to identify the sequences belonging to the *Archaea* and *Bacteria* domains and the fungi group. The taxonomic identification of the Acidobacteria community at the class taxonomic level subgroups was carried out using FOCUS software (Silva et al., 2014) with default settings, which uses a database of bacterial genomes. Initially, a table of relative abundance of bacteria occurrence was generated for each dataset at the phylum taxonomic level. Next, the 18 metagenomes were aligned using high scoring pairs in BLASTN 2.2.28 (Altschul et al., 1990) and an *E*-value threshold of 10^-5^ similarity value gene sequences against a database of Acidobacteria 16S rRNA extracted from the Ribosomal Database Project (RDP) release 11^3^ (Cole et al., 2014) for the identification of Acidobacteria subgroups. Only the best hit for each query sequence was used in the count. Relative abundances of Acidobacteria subgroups were estimated by dividing the number of sequences classified as the Acidobacteria subgroups by the total number of sequences classified as Acidobacteria per sample.

### Purified DNA Labeling and Image Scanning for GeoChip Analysis

Each of the 18 samples of the concentrated DNA was purified and labeled with Cy-3 fluorescent dye according to Wu et al. (2006). Briefly, the DNA 600 ng was mixed with random primers 300 ng mL^-1^, denatured at 99.9°C for 5 min and immediately cooled on ice. A solution containing 5 mM dAGC-TP, 2.5 mM dTTP, Klenow fragment 40 U and Cy-3 dUTP 25 nM was added to the denatured DNA, and the reaction volume was adjusted to 50 μL with H_2_O. The labeling solution was incubated at 37°C for 6 h followed by 3 min at 95°C. The labeled DNA was purified with the QIAquick Kit Qiagen, Valencia, CA, United States, and dye incorporation was confirmed with a NanoDrop spectrophotometer (NanoDrop^®^ND-1000 NanoDrop Technologies, Inc., Wilmington, DE, United States) using the absorption spectra of the standard solution for Cy-3. The labeled DNA samples were dried under vacuum and stored at -20°C until hybridization.

GeoChip v. 5.0 M, which is manufactured by Agilent (Agilent Technologies Inc., Santa Clara, CA, United States), in the 4 × 180 K format was used (Cong et al., 2015). The chip contains 167,044 distinct probes belonging to different gene categories involved in C metabolism e.g., C degradation, C fixation, methane production, N metabolism ammonification, nitrification, N fixation, S metabolism, P cycling, metal homeostasis Zn transport, secondary metabolism antibiotic, pigments, stress responses oxidative, virulence infection and other microbial genes of known function. Here, the gene sequence refers to each unique sequence targeted by GeoChip 5.0 M, and a gene family consists of all gene sequences that are assigned the same name e.g., called AceA and encode the same class of proteins. The probes originated from bacteria, archaea, fungi and viruses (both bacteriophages and eukaryotic viruses). Prior to hybridization, the labeled DNA was resuspended in 27.5 mL of DNase/RNase-free distilled water and mixed with 99.4 mL of the hybridization solution containing 2× Hi-RPM hybridization buffer (Agilent Technologies Inc., Santa Clara, CA), 10× aCGH blocking agent (Agilent Technologies Inc., Santa Clara, CA, United States), 10% formamide (JT Baker, Philipsburg, NJ, United States), 0.05 mg/mL of Cot^-1^ DNA (Agilent, Technologies Inc., Santa Clara, CA, United States) and universal standard DNA labeled with Cy5 dye 10 pM for standardization (Liang et al., 2010). The solution was denatured at 95°C for 3 min, incubated at 37°C for 30 min and hybridized with GeoChip v. 5.0M probes. The hybridization was carried out at 67°C in the presence of 10% formamide in a hybridization oven (Agilent Technologies Inc., Santa Clara, CA, United States) for 24 h. After hybridization, the slides were washed using Agilent Wash Buffers 1 and 2 following the manufacturer’s protocol. Next, the gene arrays were scanned with 100% laser power and 75% photomultiplier tube using a NimbleGen MS200 microarray scanner (Roche NimbleGen, Madison, WI, United States). The image data were extracted using Agilent Feature Extraction software v. 2.6. The alignment of points and determination of signal intensity were performed with Agilent Feature Extraction software v. 11.5 (Agilent Technologies Inc., Santa Clara, CA, United States). Data were submitted to the Microarray Data Manager available at the Institute for Environmental Genomics website http://www.ou.edu/content/ieg/tools/data-analysis-pipeline.html and analyzed using the following parameters: (i) removal of points with a signal intensity lower than 1.3 or with a signal-to-noise ratio SNR below 2.0 SNR = spot signal intensity - background mean/background SD; (ii) removal of non-representative data i.e., singletons, or positive probes in only one sample from an experimental group using the cutoff of group 1 by default; (iii) normalization performed by dividing the signal intensity of each point by the mean of the universal standard spots and then by the mean signal intensities in each sample i.e., mean ratio approach.

### Quantification of Gene Families Encoding for C Degradation Using Acidobacteria-Derived Probes

Quantification of gene families encoding for C degradation using Acidobacteria-derived probes was performed based on data obtained with GeoChip analysis for each of the 18 DNA samples. Among the 167,044 probes available in GeoChip v.5.0M, there are 7,592 probes belonging to the gene families with the “carbon degradation” function. Of this total, 100 probes with the “carbon degradation” function originated from Acidobacteria (Examples: *Solibacter usitatus* Ellin6076, *Acidobacterium capsulatum* ATCC51196 and *Granulicella mallensis* MP5ACTX8, among others). These 100 probes hybridize to 16 gene families xylanase, ara (alpha - L- arabinofuranosidase), xyla (xylose isomerase), endoglucanase, cellobiase, RgaE (acetylesterase), pme (pectinesterase), pectinase, cda (alpha amylase), glucoamylase, pula (alpha-1,6-glucosidade), amyA (alpha amylase), AceA (isocitrate liase), AceB (malato synthase), chitinase and acetylglucosaminidase from the category “carbon cycle,” subcategory 1 “carbon degradation,” subcategory 2 “hemicellulose,” “cellulose,” “pectin,” “starch,” “glyoxalate cycle” and “chitin.” In this study, only the hybridization signals with these 100 probes were considered for quantification of gene families encoding for C degradation using Acidobacteria-derived probes in the different DNA samples from the three different treatments.

### Statistical Analyses of Taxonomic and Functional Data and Soil Chemical Factors

Analysis of variance ANOVA and Tukey’s test were applied to the taxonomic Acidobacteria subgroups and functional gene families associated with C degradation data and soil chemical factors using STATISTICA software v. 13 (StatSoft Inc., Palo Alto, CA, United States). The explicit relationships among these variables were examined by constrained ordination generated by redundancy analysis RDA. The RDA was performed using CANOCO Software version 4.5 (Ter Braak and Smilauer, 1998) based on the Monte Carlo permutation test at 5% significance.

### Construction of Integration Networks of Taxonomic and Functional Data and Soil Chemical Factors

To visualize connections among the different Acidobacteria subgroups, functional genes related to carbon degradation and soil chemical factors, co-occurrence network was constructed. *Spearman* rank’s correlation coefficients were calculated (*P* < 0.05) between: (i) relative abundance of Acidobacteria subgroups and soil chemical factors, (ii) relative abundance of Acidobacteria and functional genes, and (iii) functional genes and soil chemical factors using SigmaPlot software v.14.0 SYSTAT (Software Inc., CA, United States). *Spearman* rank’s correlation coefficients from all dataset combinations with *P* < 0.05 were used to network construction. The topology number of layers, units in each layer, training algorithm parameters and activation functions were determined (Brandes, 2001; Blondel et al., 2008). The network constructed was integrated to the different application modules and analyzed using *Gephi* software (Bastian et al., 2009), which has a graphical interface.

## Results

### Soil Chemical Factors

The addition of vinasse in combination with N fertilizer at the doses used in this study promoted significant changes (*P* ≤ 0.05) in the levels of most soil chemical factors (Table 1). In particular, non-acid cations provided by the organic residue, K, Mg and the values of SB, CEC and BS were higher at 150 days in comparison with 7 days after the addition of fertilizers in soil amended with N and vinasse. In turn, the H + Al value decreased and pH increased in this circumstance. The total N, total C and OM values also increased in NV treatment at 7 to 150 days of experiment. However, OM revealed lower values for all treatments at 150 days than at 7 days after addition of fertilizers (Table 1). The S, B, Mn, and Zn levels were high to that of the treatments that did not receive vinasse at 150 days after the addition of fertilizer.

**Table 1:** Chemical compounds present in the soil treatments without nitrogen fertilization (C), with nitrogen fertilization (N) and nitrogen fertilization combined with vinasse (NV) collected at seven (T7) and one hundred and fifty (T150) days after the start of the experiment.

Physicochemical factors							Statistics								
	T7			T150			T7			T150			T7 vs T150		
	C	N	NV	C	N	NV	C vs. N	C vs. NV	N vs. NV	C vs. N	C vs. NV	N vs. NV	C	N	NV
C g.Kg^-1^	2.24^a^ ± 0.04^b^	2.29 ± 0.03	2.59 ± 0.07	2.24 ± 0.05	2.29 ± 0.03	2.59 ± 0.07		0.001	0.001		0.001	0.001			
C:N %	12.0 ± 0.30	11.9 ± 0.55	9.93 ± 0.52	12.0 ± 0.37	12.0 ± 0.55	9.93 ± 0.52		0.005	0.006		0.005	0.006			
N g.Kg^-1^	0.18 ± 0.00	0.19 ± 0.00	0.26 ± 0.02	0.18 ± 0.00	0.19 ± 0.07	0.26 ± 0.02		0.001	0.002		0.001	0.002			
pH	5.23 ± 0.06	5.23 ± 0.15	5.60 ± 1.09	5.50 ± 0.10	5.10 ± 0.00	5.80 ± 0.10		0.008	0.008	0.003	0.010	0.000	0.016		0.025
OM g.dm^-3^	38.3 ± 1.53	36.3 ± 0.58	39.7 ± 3.79	32.3 ± 1.53	32.0 ± 1.00	35.7 ± 0.57					0.024	0.016	0.008	0.003	
P mg.dm^-3^	61.0 ± 17.7	99.6 ± 12.6	90.0 ± 12.1	259 ± 94.9	248 ± 83.5	213 ± 102.0	0.038						0.023	0.038	
S mg.dm^-3^	6.33 ± 1.53	11.7 ± 0.58	184 ± 16.5	18.3 ± 7.64	17.3 ± 3.51	237.3 ± 22.3		0.000	0.000		0.000	0.000			0.028
K mmolc.dm^-3^	1.23 ± 0.06	1.13 ± 0.06	11.4 ± 0.92	3.60 ± 0.50	9.93 ± 2.72	39.0 ± 12.0		0.000	0.000		0.002	0.005	0.001	0.005	0.014
Ca mmolc.dm^-3^	54.0 ± 2.64	55.0 ± 1.00	48.7 ± 3.50	71.0 ± 2.64	56.0 ± 16.6	66.3 ± 10.1							0.002		0.046
Mg mmolc.dm^-3^	16.7 ± 1.52	17.3 ± 0.57	18.7 ± 0.58	17.3 ± 0.60	14.0 ± 1.73	22.3 ± 2.08					0.020	0.002		0.034	0.043
H+Al mmolc.dm^-3^	43.7^a^ ± 2.88^b^	44.0 ± 5.19	34.0 ± 0.00	32.0 ± 1.73	39.3 ± 2.30	25.0 ± 3.00		0.032	0.028	0.023	0.028	0.001	0.004		0.007
SB mmolc.dm^-3^	71.5 ± 3.94	73.3 ± 1.92	79.2 ± 4.85	91.8 ± 3.36	79.8 ± 18.4	128 ± 6.00					0.019	0.005			0.001
CEC mmolc.dm^-3^	115 ± 6.22	117 ± 3.42	113 ± 4.90	124 ± 4.53	119 ± 20.3	153 ± 6.75						0.037			0.001
BS %	61.7 ± 1.15	62.3 ± 3.21	70.0 ± 1.00	74.0 ± 1.00	66.7 ± 4.93	84.0 ± 1.50		0.006			0.019	0.001	0.000		0.000
B mg.dm^-3^	0.24 ± 0.04	0.22 ± 0.00	0.14 ± 0.03	0.29 ± 0.03	0.28 ± 0.02	0.37 ± 0.07		0.015						0.022	0.007
Cu mg.dm^-3^	0.93 ± 0.06	0.93 ± 0.06	0.93 ± 0.12	0.83 ± 0.06	0.93 ± 0.12	0.90 ± 0.20									
Fe mg.dm^-3^	37.0 ± 3.60	37.0 ± 4.00	48.3 ± 19.6	27.7 ± 2.08	38.0 ± 4.36	29.3 ± 7.51							0.018		
Mn mg.dm^-3^	7.40 ± 0.50	7.47 ± 1.12	21.0 ± 3.78	6.27 ± 0.81	9.37 ± 1.04	21.0 ± 11.0		0.001							
Zn mg.dm^-3^	2.00 ± 0.50	1.70 ± 0.10	2.26 ± 1.48	10.9 ± 4.67	10.8 ± 4.90	47.0 ± 26.3		0.001					0.030	0.032	0.042

*C, treatment without the addition of nitrogen fertilizer; N, treatment with nitrogen addition at the dose of 60 kg ha^-*1*^; and NV, treatment with nitrogen fertilization at the dose of 60 kg ha^-*1*^ and the addition of vinasse at a rate of 0.06 L kg^-*1*^. ^*a*^Average for each of three replicates soil. ^*b*^Standard deviations of the average for each of three replicates soil. The analyzes were performed at seven (T7) and one hundred and fifty days (T150) after the beginning of the experiment in a greenhouse. Analysis of variance and Tukey’s HSD test two-pair combination with a significance level of *P* ≤ 0.05. Blank spaces indicate that the values were not significant.*

### Relative Abundance of Acidobacteria Subgroups

The taxonomic identification of soil metagenome sequences resulted in the detection of 18 different Acidobacteria classes (Table 2) from a total of 26 subgroups – 1 to 8 according to Hugenholtz et al. (1998), subgroups 9 to 11 according to Zimmermann et al. (2005), and subgroups 12 to 26 according to Barns et al. (2007) – which demonstrates good representativeness of Acidobacteria in the soil used in this study in comparison with other tropical soils from previous studies (Navarrete et al., 2013b, 2015a,b; Catão et al., 2014). Most of the subgroups showed a decrease in abundance in the NV treatment; however, the differences between sampling times T7 and T150 were not statistically significant (Table 2). Consistent with previous studies, Gp4 was the most abundant class in the treatment that received vinasse while Gp9 and Gp18 were the least represented in the NV treatment.

**Table 2:** Abundance of Acidobacteria subgroups relative to total Acidobateria community in the soil mesocosms in the treatments without nitrogen fertilization (C), with nitrogen fertilization (N) and with nitrogen fertilization combined with vinasse (NV) over 7 days (T7) and 150 days (T150).

Acidobacteria subgroups							Statistics								
	T7			T150			T7			T150			T7 vs. T150		
	C	N	NV	C	N	NV	C vs. N	C vs. NV	N vs. NV	C vs. N	C vs. NV	N vs. NV	C	N	NV
Gp1	1.80^a^ ± 1.5^b^	4.63 ± 1.8	3.69 ± 0.3	2.04 ± 1.9	3.83 ± 0.1	3.37 ± 0.7									
Gp2	6.43 ± 1.7	2.21 ± 0.6	3.42 ± 1.1	2.37 ± 2.3	3.04 ± 0.8	1.73 ± 1.1	0.014								
Gp3	6.24 ± 3.0	5.37 ± 0.6	5.42 ± 1.0	6.65 ± 2.7	5.77 ± 0.5	5.25 ± 2.2									
Gp4	9.08 ± 2.3	11.1 ± 4.0	12.1 ± 2.2	10.7 ± 2.8	6.44 ± 0.9	13.1 ± 0.3				0.05		0.007			
Gp5	1.82 ± 0.5	1.37 ± 0.5	1.92 ± 1.9	1.04 ± 1.8	1.58 ± 0.5	1.45 ± 0.9									
Gp6	13.5 ± 1.5	11.1 ± 2.1	11.5 ± 2.1	14.3 ± 5.6	12.6 ± 1.6	6.94 ± 2.3									
Gp7	7.41 ± 2.6	8.85 ± 2.1	6.45 ± 2.3	6.23 ± 2.2	4.79 ± 2.5	6.13 ± 1.2									
Gp9	1.01 ± 0.3	0.64 ± 1.1	0.12 ± 0.2	0.52 ± 0.9	1.10 ± 0.4	0.35 ± 0.3									
Gp10	7.50 ± 1.8	6.82 ± 1.4	7.98 ± 2.6	7.43 ± 4.3	9.24 ± 0.5	6.81 ± 0.9								0.05	
Gp11	0.55 ± 0.5	1.58 ± 0.5	1.06 ± 1.3	1.01 ± 1.3	0.61 ± 0.5	0.61 ± 0.6									
Gp13	0.29 ± 0.5	0.84 ± 0.2	0.51 ± 0.4	0.17 ± 0.3	1.22 ± 0.9	0.41 ± 0.4									
Gp17	9.26 ± 2.0	8.11 ± 1.7	9.95 ± 1.4	14.4 ± 5.0	13.1 ± 2.3	11.6 ± 3.3								0.04	
Gp18	0.29 ± 0.5	0.94 ± 0.9	0.88 ± 0.2	0.35 ± 0.6	0.85 ± 0.8	0.35 ± 0.3									
Gp21	1.05 ± 0.9	1.26 ± 0.5	0.42 ± 0.4	0.00 ± 0.0	1.34 ± 1.2	1.44 ± 1.1									
Gp22	7.23 ± 2.4	8.23 ± 3.0	7.26 ± 2.1	8.38 ± 3.8	7.47 ± 2.1	6.77 ± 1.9									
Gp23	4.25 ± 1.7	4.01 ± 2.0	3.78 ± 1.2	1.70 ± 1.5	4.81 ± 1.0	3.26 ± 0.6									
Gp25	4.69 ± 2.7	3.99 ± 1.0	6.73 ± 0.4	5.66 ± 1.4	5.41 ± 0.7	10.1 ± 5.4									
Gp26	4.43 ± 0.7	4.95 ± 2.2	3.22 ± 0.7	3.13 ± 3.8	2.80 ± 2.1	4.23 ± 1.7									
Others	2.16 ± 2.0	2.26 ± 0.3	2.74 ± 0.2	2.79 ± 0.7	3.33 ± 0.2	1.76 ± 0.9								0.004	

*C, treatment without the addition of nitrogen fertilizer; N, treatment with nitrogen addition at the dose of 60 kg ha^-*1*^; and NV, treatment with nitrogen fertilization at the dose of 60 kg ha^-*1*^ and the addition of vinasse at a rate of 0.06 L kg^-*1*^. ^*a*^Average for each of three replicates soil. Values are expressed as percentage. ^*b*^Standard deviations of the average for each of three replicates soil. Analysis of variance and Tukey’s HSD test two-pair combination at the significance level of *P* ≤ 0.05. Blank spaces indicate that the values were not significant.*

In treatment C, the Gp6 and Gp17 subgroups were the most abundant. However, Gp6 showed a decrease in abundance of more than 50% in treatment NV, whereas Gp17 significantly increased its abundance in the treatments that received N fertilization N and NV. For subgroup Gp2, N fertilization does not seem to favor the occurrence of representatives of this Acidobacteria subgroup in the soil because their abundance was reduced in the N and NV treatments. The Gp13 subgroup showed low representativeness in the C treatment but was responsive to N fertilization and showed increased abundance in the N treatment but not in the NV treatment.

### Gene Families Associated With Carbon Degradation

The functional subcategories with the highest number of Acidobacteria-derived probes are “starch” and “hemicellulose” (Table 3). The 18 soil genomic DNA samples from this study hybridized with 16 gene families covered by the Acidobacteria-derived probes available in GeoChip v.5.0M – xylanase, ara, xyla, endoglucanase, cellobiase, RgaE, pme, pectinase, cda, glucoamylase, pula, amya, AceA, AceB, chitinase, and acetylglucosaminidase. The highest number of hybridizations occurred with the subcategory “starch,” followed by the subcategory “chitin” (Table 4), and the lowest number occurred with the subcategory “pectin.”

**Table 3:** Total number of probes related to the “carbon degradation” function and the subcategories in the treatments and corresponding number of probes derived only from Acidobacteria.

	T7			T150			Probes	
Carbon degradation	C	N60	NV	C	N60	NV	Total	Acidobacteria
*Total*	5002.0^a^ ± 386.3^b^	5472.6 ± 95.3	5379.1 ± 147.5	4738.2 ± 848.5	4896.0 ± 398.3	4936.7 ± 219.5	7592	103
**Gene subcategory 2**								
Starch	1717.9 ± 27.4	1877.9 ± 13.4	1847.6 ± 65.9	1635.9 ± 39.7	1692.9 ± 34.4	1732.4 ± 24.3	2674	37
Camphor	7.4 ± 0.6	8.0 ± 0.1	8.0 ± 0.1	7.2 ± 1.1	7.5 ± 0.5	7.4 ± 0.6	7	0
Cellulose	367.6 ± 7.4	411.4 ± 2.4	402.3 ± 18.1	340.9 ± 6.7	355.6 ± 4.0	353.0 ± 5.3	562	3
Chitin	741.4 ± 12.9	816.0 ± 5.9	808.4 ± 35.9	697.3 ± 19.0	719.3 ± 15.7	728.9 ± 12.9	1195	18
Cyanide	16.3 ± 0.3	18.1 ± 0.3	18.1 ± 0.8	14.6 ± 0.5	16.0 ± 0.3	15.7 ± 0.7	23	0
Cutin	134.6 ± 5.5	142.5 ± 3.5	143.3 ± 2.0	128.7 ± 16.3	128.6 ± 4.3	129.3 ± 3.7	157	0
Phospholipids	55.5 ± 0.9	61.9 ± 1.6	62.6 ± 4.0	50.7 ± 1.5	54.0 ± 0.4	52.4 ± 1.3	94	0
Glyoxylate cycle	405.0 ± 6.7	445.4 ± 3.7	435.5 ± 13.9	389.2 ± 7.6	405.4 ± 2.4	402.5 ± 4.4	595	3
Hemicellulose	620.5 ± 5.9	670.2 ± 3.9	663.5 ± 23.5	587.3 ± 16.1	608.1 ± 8.9	611.0 ± 7.9	947	23
Inulin	8.2 ± 0.2	9.2 ± 0.2	9.4 ± 0.6	7.7 ± 0.3	8.3 ± 0.0	8.3 ± 0.2	11	0
Lactose	11.6 ± 0.2	12.9 ± 0.6	10.6 ± 0.2	9.9 ± 0.3	10.3 ± 0.2	10.4 ± 0.3	25	0
Lignin	182.8 ± 3.3	202.5 ± 3.6	193.0 ± 5.8	171.4 ± 3.5	176.2 ± 1.6	174.0 ± 2.1	273	0
Others	10.7 ± 0.3	12.5 ± 0.7	11.5 ± 0.9	8.4 ± 0.7	9.6 ± 0.2	9.4 ± 0.5	23	0
Pectin	447.0 ± 6.3	482.0 ± 5.6	470.7 ± 13.0	429.9 ± 11.2	431.9 ± 8.7	440.9 ± 4.2	645	19
Protein	19.9 ± 0.4	22.6 ± 1.1	20.6 ± 0.4	17.9 ± 0.3	19.3 ± 0.7	18.2 ± 0.6	36	0
Tannins	20.4 ± 0.3	22.4 ± 0.4	21.0 ± 0.9	17.5 ± 0.2	19.7 ± 0.3	18.1 ± 0.1	29	0
Terpenes	90.5 ± 1.4	99.9 ± 0.6	98.6 ± 3.7	86.4 ± 1.8	90.2 ± 0.9	87.5 ± 0.6	112	0
Valin/Lignin	144.4 ± 1.6	157.0 ± 1.0	154.3 ± 5.4	137.1 ± 3.3	143.2 ± 1.1	137.2 ± 2.3	184	0

*C, treatment without the addition of nitrogen fertilizer; N, treatment with nitrogen addition at the dose of 60 kg ha^-*1*^; and NV, treatment with nitrogen fertilization at the dose of 60 kg ha^-*1*^ and the addition of vinasse at a rate of 0.06 L kg^-*1*^. ^*a*^Average for each of three replicates soil. ^*b*^Standard deviations of the average for each of three replicates soil.*

**Table 4:** Signal intensities of the families of carbon degradation genes obtained using GeoChip v. 5.0M from Acidobacteria and hybridized with DNA sampled at seven (T7) and one hundred and fifty (T150) days after vinasse application.

Carbon degradation genes	T7			T150			Statistics								
							T7 vs. T150			T7			T150		
	C	N	NV	C	N	NV	C	N	NV	C vs. N	C vs. NV	N vs. NV	C vs. N	C vs. NV	N vs. NV
**Glyoxalato cycle**															
AceA	198.3^a^ ± 10.6^b^	210.9 ± 4.3	205.7 ± 4.6	190.1 ± 21.3	192.9 ± 6.0	191.1 ± 1.5		0.01	0.01						
AceB	313.3 ± 16.6	331.1 ± 5.7	329.8 ± 2.4	305.9 ± 28.3	311.0 ± 7.5	303.6 ± 4.7		0.02	0.00						
**Chitin**															
acetylglucosaminidase	347.3 ± 22.8	373.3 ± 4.1	367.6 ± 3.9	340.9 ± 36.9	343.6 ± 14.7	338.1 ± 5.4		0.03	0.00						
chitinase	722.1 ± 45.2	764.9 ± 5.8	758.6 ± 7.4	700.0 ± 69.0	712.0 ± 17.8	696.8 ± 4.1		0.01	0.00						
**Starch**															
amyA	1953.0 ± 116.5	2084.1 ± 15.2	2049.0 ± 24.8	1885.1 ± 200.7	1901. ± 70.3	1895.7 ± 13.6		0.01	0.00						
cda	148.6 ± 11.1	158.0 ± 2.5	156.3 ± 3.1	146.5 ± 16.1	147.0 ± 3.9	140.5 ± 3.2		0.01	0.00						
glucoamylase	107.8 ± 5.8	113.1 ± 0.4	111. 7 ± 0.4	103.67 ± 9.7	103.0 ± 7.1	104.4 ± 1.9			0.00						
pula	120.4 ± 7.8	127.3 ± 2.6	126.6 ± 2.4	116.2 ± 13.4	115.4 ± 7.5	115.6 ± 2.0			0.00						
**Hemicellulose**															
ara	273.5 ± 14.7	289.1 ± 2.2	285.7 ± 1.9	270.8 ± 19.9	272.2 ± 8.0	267.2 ± 3.6		0.02	0.00						
xyla	176.0 ± 10.1	185.5 ± 1.3	183.8 ± 4.7	173.5 ± 16.9	171.2 ± 6.1	170.3 ± 2.5		0.02	0.01						
xylanase	273.4 ± 18.3	291.2 ± 2.8	286.1 ± 2.2	266.1 ± 25.0	270.4 ± 12.8	263.0 ± 2.6		0.05	0.00						
**Cellulose**															
cellobiase	229.6 ± 14.3	245.2 ± 2.1	239.0 ± 3 1.3	221.3 ± 24.9	223.1 ± 8.3	223.1 ± 3.0		0.01	0.00						
endoglucanase	167.0 ± 11.1	177.5 ± 1.6	175.0 ± 1.7	164.0 ± 14.7	165.5 ± 6.0	161.6 ± 2.3		0.03	0.00						
**Pectin**															
pectinase pectate_liase	96.27 ± 6.4	101.9 ± 1.3	101.3 ± 2.4	92.91 ± 8.7	94.17 ± 4.3	94.4 ± 2.1		0.04	0.02						
pmE	95.20 ± 5.0	100.2 ± 2.9	100.3 ± 1.4	94.26 ± 7.9	95.09 ± 5.3	94.9 ± 1.8			0.01						
RgaE	118.2 ± 6.5	124.1 ± 1.5	122.3 ± 3.0	114.7 ± 10.2	116.5 ± 1.6	116.3 ± 1.0		0.00	0.03						

*C, treatment without the addition of nitrogen fertilizer; N, treatment with nitrogen addition at the dose of 60 kg ha^-*1*^; and NV, treatment with nitrogen fertilization at the dose of 60 kg ha^-*1*^ and the addition of vinasse at a rate of 0.06 L kg^-*1*^. ^*a*^Average for each of three replicates soil. ^*b*^Standard deviations of the average for each of three replicates soil. Analysis of variance and Tukey’s HSD test two-pair combination at a significance level of *P* ≤ 0.05. Blank spaces indicate that the values were not significant.*

A significant decrease (*P* ≤ 0.05) in the signal intensity of the hybridizations between the soil genomic DNA and Acidobacteria derived probes belonging to the gene families associated with C degradation was observed in the NV treatment when comparing T7 and T150. In the N treatment, most gene families showed a significant decrease (*P* ≤ 0.05) in the hybridization signal intensity in the same period (T7 and T150) (Table 4). In the C treatment, significant differences were not observed in the hybridization signal intensity.

### Relationship Among the Relative Abundance of Acidobacteria Subgroups, Gene Families Associated With C Degradation and Soil Chemical Characteristics

**FIGURE 1:**
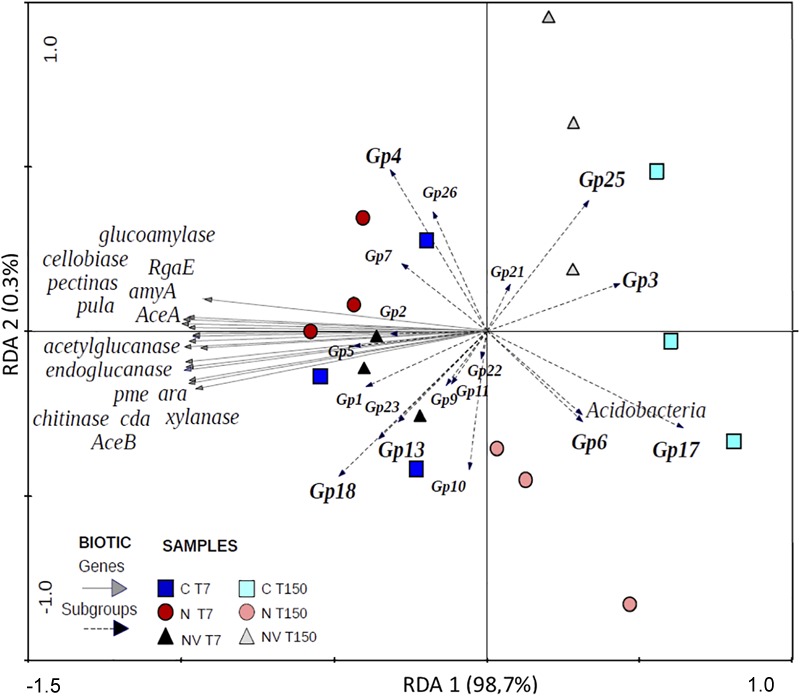
Constrained ordination diagram for sample plots in the first two redundancy analysis (RDA) axes based on the soil physicochemical factors of the different soil treatments and their relationship with the Acidobacteria subgroups and carbon degradation gene families.

The RDA revealed two distinct groups according to the sampling times (Figure 1). The abundance of the Gp3, Gp6, Gp17, Gp21, and Gp25 subgroups was positively related to the chemical characteristics of the soil samples collected at T150. The abundance of most of the subgroups identified in this study and all the gene families analyzed were positively related to the chemical characteristics of soil samples collected at T7.

**FIGURE 2:**
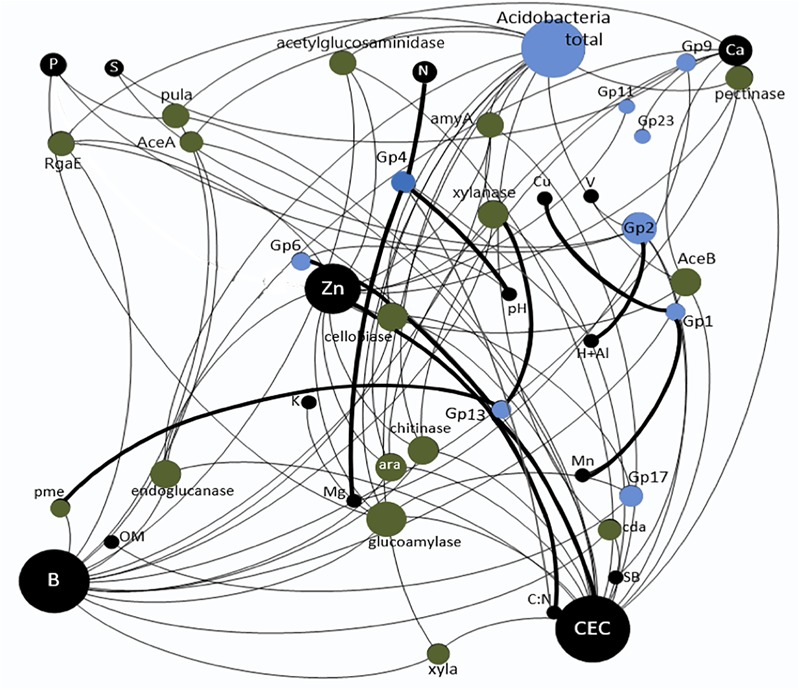
Correlation among the soil chemical factors, different Acidobacteria subgroups and quantified carbon degradation gene families in the treatments with nitrogen fertilization and vinasse application. The size of the circles corresponds to the number of interactions. The thicker lines correspond to positive interactions, and the thinner lines correspond to negative interactions.

The combination of the three datasets taxonomic data, functional data and soil chemical factors for the construction of the neural network based on all correlations significant or not in *Spearman’s* non-parametric test resulted in a network with a total of 42 nodes and 85 edges and a modularity of -0.174 (Figure 2). The constructed network improved the visualization and interpretation of the theoretical relationships among the Acidobacteria subgroups, gene families and soil chemical factors beyond those revealed by the RDA.

Based on the network, the greatest number of observed interactions occurred between the Acidobacteria subgroups and the soil chemical factors, most of which were negative. The chemical factors with the greatest number of interactions with the gene families and the subgroups were CEC, B and Zn. Only the Gp3 and Gp6 subgroups presented positive interactions with Zn and CEC. *Spearman’s* correlation analysis (Supplementary Table S1) showed a significantly positive correlation between Gp6 and the C:N ratio. The Gp2 subgroup presented the greatest number of interactions in the network and was the only subgroup to interact positively with H + Al. *Spearman’s* correlation analysis showed that this subgroup was negatively correlated with S and BS (Supplementary Table S1). The only subgroup that showed significant negative correlations with all C degradation gene families was Gp17 (Supplementary Table S1). However, this subgroup was significantly positively correlated with P, BS, V, B, and Zn (Figure 1 and Supplementary Table S1). The only class that showed positive correlations with the gene families involved with C degradation in the network was Gp13, which was correlated with “xylanase” and “pme” belonging to the subcategories “hemicellulose” and “pectin,” respectively. However, *Spearman’s* correlation analysis indicated that this subgroup was significantly positively correlated with 11 of the 16 gene families evaluated (Supplementary Table S2). With a similar profile based on *Spearman’s* correlation analysis, Gp18 also showed a positive correlation with most gene families (Supplementary Table S2); however, it showed a negative correlation with Ca and CEC (Supplementary Table S1). Subgroup Gp1 showed a significant positive correlation with Cu and Mn (Figure 1), and *Spearman’s* correlation analysis indicated that this subgroup was correlated with Fe (Supplementary Table S1). The Acidobacteria subgroups Gp4 and Gp6 were positively correlated with the soil pH (Supplementary Table S1 and Figure 1). Nine Acidobacteria subgroups revealed significant correlation among them (Supplementary Table S3).

## Discussion

One of the most ubiquitous and abundant bacterial phyla in the soil environment still has large knowledge gaps regarding its role in soil. Analyses based on genomic DNA from environmental samples have provided relevant information on the metabolic potential of Acidobacteria (Liles et al., 2003; Lee et al., 2008; Faoro et al., 2012; Lin et al., 2019) and led to alternative methods of culture-dependent techniques, which can provide a better understanding of the ecology of this recalcitrant bacterial phylum in culture medium. Such analyses based on culture-independent techniques have provided insights into the role of Acidobacteria in the soil environment. This study combines genomic, chemical, statistical and computational analyses to obtain information about the dynamics of Acidobacteria subgroups and their potential for C degradation in soils under sugarcane in a tropical region. Previous studies on tropical Amazonian soils with Acidobacteria are consistent with the results presented here, in which different lifestyles were observed for the different subgroups (Navarrete et al., 2010, 2013b, 2015b) except for subgroups Gp6 and Gp17. However, the functional aspects of the Acidobacteria subgroups have not been studied yet in tropical soils.

Navarrete et al. (2010) showed that a high abundance of the subgroup Gp6 occurs in Amazonian Dark Earth (ADE), which presents high levels of OM and nutrients and a pH about 5.0. The Gp6 subgroup was also highly abundant in 27 pasture soils in Germany, where the pH, N, P and temperature values were high and the C:N ratio was low compared with that of forest soils in the region Naether et al. (2012). Although the Gp6 subgroup showed a positive correlation with the Zn contents, CEC and C:N ratio, a decrease in the abundance of this subgroup was observed in the NV treatment, which presented chemically similar conditions as ADE and European pasture soils. These observations indicate that the Gp6 subgroup has a preference for soils with pH below 5.5 (Lin et al., 2019), which justifies its negative correlation with the pH in this study as well as its high abundance in the C treatment pH 5.2 to 5.5 and decreased abundance in the NV treatment pH 5.8. Authors have suggested that this subgroup may be positively or negatively correlated with soil pH (Chan et al., 2006; Mukherjee et al., 2014), which shows that the behavior of the same Acidobacteria subgroup can vary in different types of soil (Naether et al., 2012). Additionally, studies have explored the interactions of the Gp6 subgroup with bacteria from other phyla and even protozoa (Spring et al., 2000; Naether et al., 2012). However, these studies have not provided conclusive results for the possible ecological interactions.

The abundance of subgroup Gp17 has been positively correlated with pH in pasture soils (Naether et al., 2012; Navarrete et al., 2015b). Although the abundance of subgroup Gp17 was not correlated with the soil pH in this study, it was correlated with several chemical factors in soil treatment that received N fertilizer combined with vinasse. In ADE with high N levels and pH values approximately 5.0, this group was not very abundant (Navarrete et al., 2010), suggesting that the occurrence of Gp17 can be modulated by other chemical factors, such as P, BS, V, B, and Zn. Although the Gp17 subgroup has the potential to become one of the most abundant Acidobacteria subgroups in soils under sugarcane supplemented with vinasse, it probably does not have genes related to the degradation of hemicellulose, cellulose, pectin, starch and chitin and/or participation in the glyoxalate cycle according to the results of this study. This result suggests that C degradation metabolic pathways different from those analyzed here or ecological interactions with other microorganisms must occur to obtain the required C sources for their development.

The predominance of the Gp4 subgroup in soil under sugarcane supplemented with vinasse is corroborated by a previous study by Omori et al. (2016). The results of this study also showed that a longer period after vinasse application contributes to the increased abundance of the Gp4 subgroup. Navarrete et al. (2010) also showed a predominance of the Gp4 subgroup when ADE is supplemented with biochar or biocarbon. Considering that biocarbon constitutes a hotspot and presents high Ca, Mg, and Zn amounts, pH values that reach 10.0, microaerophilic conditions and higher temperatures than the adjacent soil, the increased abundance of this subgroup in the vinasse treatment suggests that soil microhabitats in fields treated with this residue provide adequate physical and chemical conditions for the development of these bacteria (Kuzyakov and Blagodatskaya, 2015). Studies have indicated that the application of vinasse for a short period of time in the soil can reduce the water infiltration rate and total porosity due to the cementing effect of OM (Dalri et al., 2010; dos Santos et al., 2017), suggesting that the addition of vinasse may alter the soil aggregate structure. *Chloracidobacterium thermophilum*, a representative of subgroup Gp4 isolated from hot springs, is microaerophilic and presents optimal growth at a temperature of 5°C and pH ranging from 5.0 to 7.0 (Tank and Bryant, 2015). *Pyrinomonas methylaliphatogenes* K22T is another isolate from New Zealand hot springs that has been grown in low nutrient culture medium, and it is moderately acidophilic with an optimal growth pH of 6.5 (Lee et al., 2015). Wust et al. (2016) detected exopolysaccharides (EPS) production in Gp4 isolates from African savanna soil, which is interesting because EPS can be used by microorganisms to remain attached to soil particles. Although the GP4 subgroup has been described as one of the most versatile in the use of carbohydrates because it can use C from cellobiose, sucrose, maltose, chitins, carboxymethyl cellulose and microcrystalline cellulose (Foesel et al., 2013; Huber et al., 2014; Lee et al., 2015), the analyses performed in this study did not reveal interactions of this subgroup with the families involved in C degradation. The large number of hybridizations between the Acidobacteria-derived probes and the gene families associated with “chitin” degradation can be explained by the fact that the Gp4 subgroup, which is abundant in treatments with NV and containing hydrolase (Huber et al., 2014), has the potential to use the C found in the cell wall of fungi used in the fermentative process that result in the vinasse. Nakamura et al. (2014) performed a prospective study of isolates that degrade aromatic hydrocarbons in ADE and enriched culture media with C from lignocellulose but did not obtain any Acidobacteria isolates. Kielak et al. (2016) warned against premature conclusions about the ability of Acidobacteria to degrade these polysaccharides since there are discrepancies between the information contained in the genomes of cultured representatives and the result of physiological tests with such representatives.

The subgroups Gp13 and Gp18 presented the highest number of positive correlations with the gene families related to C degradation, especially those involved in hemicellulose degradation. However, both subgroups presented low abundance in the treatment containing vinasse, which indicates that the physical and chemical conditions imposed by the addition of this organic residue to the soil has a negative effect on the occurrence of these subgroups. The negative correlations between the Gp18 subgroup and the Ca and CEC levels indicate that representatives of these subgroups may benefit from a lower availability of nutrients, which is inconsistent with the conditions generated via the addition of vinasse to soil. Navarrete et al. (2015b) observed that the abundance of subgroup Gp18 increased in pasture soil, which presents higher pH and nutrient availability relative to forest soils. Although the soil subjected to vinasse application in this study had chemical characteristics very similar to that of pasture soils (Naether et al., 2012; Navarrete et al., 2015b), subgroup Gp18 was the least abundant in the NV treatment. Physical factors that can be altered in the presence of vinasse and in the pasture, such as soil moisture and aeration (Dedecek et al., 2000; Silva Filho et al., 2010; dos Santos et al., 2017), may have influenced the occurrence of the Gp18 subgroup. In the conversion of forest to pasture, Gp13 decreases significantly (Navarrete et al., 2015b), suggesting that the ecological role of these microorganisms in natural environments is important. Unfortunately, anthropogenic impacts on the soil environment, such as the addition of agroindustrial residues and pasture establishment, may promote the reduced abundance of subgroups with relevant functions in C degradation.

This study also provided additional evidence on the metabolic potential of Gp2 regarding Al. Similar to observations in soil from the Amazon forest, Atlantic forest and Cerrado Brazilian Savanna (Navarrete et al., 2013b; Catão et al., 2014; de Carvalho et al., 2016), this subgroup was positively associated with H + Al.

Recent studies on the phylum Acidobacteria have suggested that its subgroups differ with respect to their lifestyle, with some showing oligotrophic behavior and others showing copiotrophic behavior (Kielak et al., 2016; Yao et al., 2017), and the behavior associated with the lifestyle may even vary for the same subgroup in different soil types as mentioned above. In this study, the gene families associated with C degradation and most of the Acidobacteria subgroups were positively correlated with the soil chemical characteristics at T7, in which the lowest N, C, Ca, Mg, K levels were obtained (Figure 1). Olander and Vitousek (2000) evaluated the chitinase enzyme activity in Hawaiian soils with different ages and N levels, and the authors concluded that enzymatic activity was inhibited in older soils with higher N levels while chitinase production was activated in younger soils with lower total N levels. Chitinase is one of the most abundant enzymes in tropical soils because these soils contain a large amount of fungi with chitinous cell wall and invertebrates (Olander and Vitousek, 2000). In soils supplemented with vinasse, a residue resulting from the fermentation process, the amount of yeast is high. In this study, the high availability of N in the treatments observed in T150 may have altered the enzymatic metabolism associated with C in Acidobacteria, with carbon dioxide and nitrous oxide emissions increased in the first days after application of the fertilizers as revealed by Navarrete et al. (2015a) when assessing the same experiment and sugarcane soil. Access to OM occurred before T150, when the stocks are significantly reduced (Table 1). If the addition of N as fertilizer can reduce the decomposition of recalcitrant C (Craine et al., 2007), then the members of the bacterial community that act as decomposers of this C fraction would be promoted, which suggests that Acidobacteria members that increase in abundance in soils that receive N fertilization in combination with vinasse such as the Gp4 subgroup may be able to use different sources of C and adapt to variations in the physicochemical conditions of the environment. In this sense, Gp4 would be able to change its lifestyle from oligotrophic to copiotrophic depending on the selective pressure of the environment. Morphologically, the Gp4 isolates from African savanna soil showed cytoplasmic extensions, indicating adaptations for nutrient uptake in nutrient-poor environments (Wust et al., 2016). In addition, these isolates contain genes encoding thermophilic hydrolase enzymes (Huber et al., 2014).

In soils fertilized with vinasse, the pH seems to have little influence on the general distribution of Acidobacteria subgroups. Recent studies involving Acidobacteria have questioned the strong correlation between these bacteria and pH because whether this correlation is based on a direct causal relationship or the covariation among different soil chemical factors remains unclear (Kielak et al., 2016). Suleiman et al. (2018) also suggest that the application of organic residues to soil results in an increase in the abundance of microbial groups related to the N cycle. Physical factors altered by the application of vinasse may influence the occurrence and frequency of Acidobacteria subgroups, and such factors include temperature and/or organisms that possibly interact with Acidobacteria. Future studies should include analyses of soil physical factors in addition to chemical factors as well as of aspects related to the interactions between Acidobacteria subgroups and the biotic factors of their environment. Moreover, the lack of isolates of most Acidobacteria subgroups increases the difficulty of conducting physiological tests to validate genetic predictions, which is a limiting factor for advancing our understanding of the role of Acidobacteria in ecosystems. Genomic and physiological studies with isolates of the Gp4 subgroup can contribute to a better understanding of the role of Acidobacteria in C degradation in the soil and could provide new possibilities for the application of the biotechnological potential of these bacteria favored in sugarcane soil enriched with N and vinasse.

## Conclusion

The addition of N and vinasse to soil under sugarcane can increase the availability of nutrients in this environment, especially Ca, Mg, K, Al, B, and Zn, whose increased levels in the soil were related to decrease in the abundance of Acidobacteria subgroups and gene families associated with C degradation. The Gp13 and Gp18 subgroups, which are positively associated with C degradation, did not show adaptive success to the physical-chemical conditions imposed by the addition of N and vinasse in the soil, suggesting that changes resulting from this agricultural soil management practice can affect C metabolism in Acidobacteria.

## Data Availability

The datasets generated for this study can be found in the Metagenomics Rapid Annotation (MG-RAST) server under the “Metagenome of sugarcane soil - CENA/USP” project, Accession Numbers from 4582104.3 to 4582153.3.

## Author Contributions

AN and ST conceived and designed the study. AN, RR, and ST conducted the research. MdC, GS, RE, and AN analyzed the data. MdC and AN wrote the manuscript.

## Conflict of Interest Statement

The authors declare that the research was conducted in the absence of any commercial or financial relationships that could be construed as a potential conflict of interest.

## Footnotes

1 http://www.bioinformatics.org/

2 http://metagenomics.anl.gov

3 http://rdp.cme.msu.edu/
