# Supplementary material for: Acidobacteria Subgroups and Their Metabolic Potential for Carbon Degradation in Sugarcane Soil Amended With Vinasse and Nitrogen Fertilizers
Source: Front Microbiol. 2019 Jul 30;10:1680. doi: 10.3389/fmicb.2019.01680 (PMC6682628; doi:10.3389/fmicb.2019.01680)
Supplement: Supplementary file 1 [file Table_1.docx]

**Acidobacteria subgroups and their metabolic potential for carbon degradation in sugarcane soil amended with vinasse and nitrogen fertilizers**

**Miriam Gonçalves de Chaves^1^, Genivaldo Gueiros Z. Silva^2^, Raffaella Rossetto^3^, Robert Alan Edwards^2^, Siu Mui Tsai^1^, Acacio Aparecido Navarrete^1,4*^**

^1^Cell and Molecular Biology Laboratory, Center for Nuclear Energy in Agriculture, University of São Paulo USP, Piracicaba, SP, Brazil.

^2^Computational Science Research Center, San Diego State University, San Diego, CA, USA.

^3^São Paulo´s Agency for Agribusiness Technology, Piracicaba, SP, Brazil.

^4^Department of Environmental Sciences, Federal University of São Carlos, Sorocaba, SP, Brazil.

**Supplementary material**

**Supplementary Table S1.** *Spearman’s* correlation values r_s_ between the Acidobacteria classes and physicochemical factors quantified in the soil samples

| **Acidobacteria**  **subgroup** | OM | P | S | K | Ca | Mg | H+Al | SB | CEC | V | B | Cu | Fe | Mn | Zn | C | C/N | N | pH |  |
| --- | --- | --- | --- | --- | --- | --- | --- | --- | --- | --- | --- | --- | --- | --- | --- | --- | --- | --- | --- | --- |
|  |  |  |  |  |  |  |  |  |  |  |  |  |  |  |  |  |  |  |  |  |
|  |  |  |  |  |  |  |  |  |  |  |  | 0.6 | 0.479 | 0.45 |  |  |  |  |  |  |
| Gp1 |  |  |  |  |  |  |  |  |  |  |  | **0.008** | **0.043** | **0.059** |  |  |  |  |  |  |
|  | 0.479 |  | -0.54 |  |  |  |  | -0.48 |  |  |  |  |  |  |  |  |  |  |  |  |
| Gp2 | **0.043** |  | **0.022** |  |  |  |  | **0.043** |  |  |  |  |  |  |  |  |  |  |  |  |
|  |  |  |  |  |  |  |  |  |  |  |  |  |  |  |  |  |  |  |  |  |
| Gp3 |  |  |  |  |  |  |  |  |  |  |  |  |  |  |  |  |  |  |  |  |
|  |  |  |  |  |  |  |  |  |  |  |  |  |  |  |  |  |  |  |  |  |
| Gp4 |  |  |  |  |  | 0.592 | -0.494 |  |  |  |  |  |  |  |  |  | -0.59 | 0.56 | 0.617 |  |
|  |  |  |  |  |  | **0.01** | **0.036** |  |  |  |  |  |  |  |  |  | **0.01** | **0.015** | **0.006** |  |
| Gp5 |  |  |  |  |  |  |  |  |  |  |  |  |  |  |  |  |  |  |  |  |
|  |  |  |  |  |  |  |  |  |  |  |  |  |  |  |  |  |  |  |  |  |
| Gp6 |  |  |  |  |  |  |  |  |  |  |  |  |  |  |  |  | 0.523 | -0.518 | -0.509 |  |
|  |  |  |  |  |  |  |  |  |  |  |  |  |  |  |  |  | **0.025** | **0.027** | **0.031** |  |
| Gp7 |  |  |  |  |  |  |  |  |  |  |  |  |  |  |  |  |  |  |  |  |
|  |  |  |  |  |  |  |  |  |  |  |  |  |  |  |  |  |  |  |  |  |
| Gp9 |  |  | -0.48 |  |  | -0.47 |  |  |  |  |  |  |  |  |  |  |  |  |  |  |
|  |  |  | **0.042** |  |  | **0.046** |  |  |  |  |  |  |  |  |  |  |  |  |  |  |
| Gp10 |  |  |  |  |  |  |  |  |  |  |  |  |  |  |  |  |  |  |  |  |
|  |  |  |  |  |  |  |  |  |  |  |  |  |  |  |  |  |  |  |  |  |
| Gp11 |  |  |  |  |  |  |  |  |  |  |  |  |  |  |  |  |  |  |  |  |
|  |  |  |  |  |  |  |  |  |  |  |  |  |  |  |  |  |  |  |  |  |
| Gp13 |  |  |  |  |  |  |  |  |  |  |  |  |  |  |  |  |  |  |  |  |
|  |  |  |  |  |  |  |  |  |  |  |  |  |  |  |  |  |  |  |  |  |
| Gp17 | -0.53 | 0.707 |  |  |  |  |  | 0.505 |  | 0.5 | 0.46 |  |  |  | 0.482 |  |  |  |  |  |
|  | **0.022** | **0.001** |  |  |  |  |  | **0.032** |  | **0.03** | **0.06** |  |  |  | **0.042** |  |  |  |  |  |
| Gp18 |  |  |  |  | -0.48 |  |  |  | -0.558 |  |  |  |  |  |  |  |  |  |  |  |
|  |  |  |  |  | **0.042** |  |  |  | **0.016** |  |  |  |  |  |  |  |  |  |  |  |
| Gp21 |  |  |  |  |  |  |  |  |  |  |  |  |  |  |  |  |  |  |  |  |
|  |  |  |  |  |  |  |  |  |  |  |  |  |  |  |  |  |  |  |  |  |
| Gp22 |  |  |  |  |  |  |  |  |  |  |  |  |  |  |  |  |  |  |  |  |
| Gp23 |  |  |  |  | -0.6 |  |  | -0.608 |  | -0.5 |  |  |  |  |  |  |  |  |  |  |
|  |  |  |  |  | **0.009** |  |  | **0.007** |  | **0.03** |  |  |  |  |  |  |  |  |  |  |
| Gp25 |  |  | 0.488 | 0.559 |  |  |  |  |  |  |  |  |  | 0.487 |  |  |  |  |  |  |
|  |  |  | **0.039** | **0.016** |  |  |  |  |  |  |  |  |  | **0.039** |  |  |  |  |  |  |
| Gp26 |  |  |  |  |  |  |  |  |  |  |  |  |  |  |  |  |  |  |  |  |
|  |  |  |  |  |  |  |  |  |  |  |  |  |  |  |  |  |  |  |  |  |

*Spearman’s* correlation coefficient; Highlighted: significant at *P* ≤ 0.05

**Supplementary Table S2.** *Spearman’s* correlation values r_s_ between Acidobacteria classes and carbon degradation genes based on GeoChip v. 5.0M detection among the samples

| **Acidobacteria**  **subgroups** | AceA | AceB | acetylglucosaminidase | amyA | ara | cda | cellobiase | endoglucanase | chitinase | glucoamylase | pectinase | pme | pula | RgaE | xyla | xylanase |
| --- | --- | --- | --- | --- | --- | --- | --- | --- | --- | --- | --- | --- | --- | --- | --- | --- |
|  | -0.465 |  | -0.482 | -0.478 |  |  |  | -0.501 | -0.461 | -0.558 | -0.501 |  |  | -0.463 |  | -0.511 |
| Gp1 | **0.05** |  | **0.042** | **0.044** |  |  |  | **0.034** | **0.053** | **0.016** | **0.034** |  |  | **0.052** |  | **0.03** |
|  |  | 0.474 |  |  |  |  |  |  |  |  |  |  |  |  |  |  |
| Gp2 |  | **0.046** |  |  |  |  |  |  |  |  |  |  |  |  |  |  |
|  |  |  |  |  |  |  |  |  |  |  |  |  |  |  |  |  |
| Gp3 |  |  |  |  |  |  |  |  |  |  |  |  |  |  |  |  |
|  |  |  |  |  |  |  |  |  |  |  |  |  |  |  |  |  |
| Gp4 |  |  |  |  |  |  |  |  |  |  |  |  |  |  |  |  |
|  |  |  |  |  |  |  |  |  |  |  |  |  |  |  |  |  |
| Gp5 |  |  |  |  |  |  |  |  |  |  |  |  |  |  |  |  |
|  |  |  |  |  |  |  |  |  |  |  |  |  |  |  |  |  |
| Gp6 |  |  |  |  |  |  |  |  |  |  |  |  |  |  |  |  |
|  |  |  |  |  |  |  |  |  |  |  |  |  |  |  |  |  |
| Gp7 |  |  |  |  |  |  |  |  |  |  |  |  |  |  |  |  |
|  |  |  |  |  |  |  |  |  |  |  |  |  |  |  |  |  |
| Gp9 |  |  |  |  |  |  |  |  |  |  |  |  |  |  |  |  |
|  |  |  |  |  |  |  |  |  |  |  |  |  |  |  |  |  |
| Gp10 |  |  |  |  |  |  |  |  |  |  |  |  |  |  |  |  |
|  |  |  |  |  |  |  |  |  |  |  |  |  |  |  |  |  |
| Gp11 |  |  |  |  |  |  |  |  |  |  |  |  |  |  |  |  |
|  |  |  |  |  |  |  |  |  |  |  |  |  |  |  |  |  |
| Gp13 |  |  |  |  |  |  |  |  |  |  |  |  |  |  |  |  |
|  |  | 0.515 | 0.519 | 0.505 | 0.509 |  | 0.504 | 0.539 | 0.516 |  | 0.523 | 0.56 |  |  |  | 0.566 |
| Gp17 |  | **0.028** | **0.027** | **0.032** | **0.031** |  | **0.033** | **0.02** | **0.028** |  | **0.025** | **0.015** |  |  |  | **0.015** |
|  | -0.564 | -0.51 | -0.546 | -0.602 | -0.48 | -0.48 | -0.614 | -0.552 | -0.562 | -0.664 | -0.527 | -0.455 | -0.455 | -0.583 | -0.525 | -0.562 |
| Gp18 | **0.014** | **0.029** | **0.019** | **0.008** | **0.043** | **0.045** | **0.007** | **0.017** | **0.015** | **0.003** | **0.024** | **0.056** | **0.056** | **0.011** | **0.025** | **0.015** |
|  |  | 0.575 | 0.486 | 0.454 | 0.488 | 0.501 |  | 0.017 | 0.462 |  |  |  |  |  |  | 0.473 |
| Gp21 |  | **0.013** | **0.04** | **0.058** | **0.039** | **0.034** |  | **0.017** | **0.052** |  |  |  |  |  |  | **0.046** |
|  |  |  |  |  |  |  |  |  |  |  |  |  |  |  |  |  |
| Gp22 |  |  |  |  |  |  |  |  |  |  |  |  |  |  |  |  |
|  |  |  |  |  |  |  |  |  |  |  |  |  |  |  |  |  |
| Gp23 |  |  |  |  |  |  |  |  |  |  |  |  |  |  |  |  |
|  |  |  |  |  |  |  |  |  |  |  |  |  |  |  |  |  |
| Gp25 | -0.496 |  | -0.472 | -0.488 | -0.484 |  | -0.451 |  |  | -0.562 |  |  |  |  |  |  |
|  | **0.035** |  | **0.047** | **0.04** | **0.041** |  | **0.06** |  |  | **0.015** |  |  |  |  |  |  |
| Gp26 |  |  |  |  |  |  |  |  |  |  |  |  |  |  |  |  |

*Spearman’s* correlation coefficient; Highlighted: significant at *P* ≤ 0.05

**Supplementary Table S3.** *Spearman’s* correlation values r_s_ between the classes of Acidobacteria samples in the three treatments with level of significance *P* ≤ 0.05

|  |  | Gp1 | Gp2 | Gp3 | Gp4 | Gp5 | Gp6 | Gp7 | Gp9 | Gp10 | Gp11 | Gp13 | Gp17 | Gp18 | Gp21 | Gp22 | Gp23 | Gp25 | Gp26 | Others |
| --- | --- | --- | --- | --- | --- | --- | --- | --- | --- | --- | --- | --- | --- | --- | --- | --- | --- | --- | --- | --- |
|  | Gp1 |  |  |  |  |  |  |  |  |  |  | 0.557 |  | 0.654 |  |  |  |  |  |  |
|  |  |  |  |  |  |  |  |  |  |  |  | **0.0159** |  | **0.0032** |  |  |  |  |  |  |
|  | Gp2 |  |  |  |  | 0.597 |  |  | 0.584 |  |  |  |  |  |  |  |  |  |  |  |
|  |  |  |  |  |  | **0.009** |  |  | **0.011** |  |  |  |  |  |  |  |  |  |  |  |
|  | Gp3 |  |  |  |  |  |  |  |  |  |  |  |  |  |  |  |  |  |  |  |
|  |  |  |  |  |  |  |  |  |  |  |  |  |  |  |  |  |  |  |  |  |
|  | Gp4 |  |  |  |  |  |  |  | -0.518 |  |  |  |  |  |  |  |  |  |  |  |
|  |  |  |  |  |  |  |  |  | **0.0276** |  |  |  |  |  |  |  |  |  |  |  |
|  | Gp5 |  |  |  |  |  | -0.497 |  | 0.486 |  |  |  |  |  |  |  |  |  |  |  |
|  |  |  |  |  |  |  | **0.0355** |  | **0.04** |  |  |  |  |  |  |  |  |  |  |  |
|  | Gp6 |  |  |  |  |  |  |  |  |  |  |  |  |  |  |  |  |  |  |  |
|  |  |  |  |  |  |  |  |  |  |  |  |  |  |  |  |  |  |  |  |  |
|  | Gp7 |  |  |  |  |  |  |  |  |  |  |  |  |  |  |  |  |  |  |  |
|  |  |  |  |  |  |  |  |  |  |  |  |  |  |  |  |  |  |  |  |  |
|  | Gp9 |  |  |  |  |  |  |  |  |  |  |  |  |  |  |  |  |  |  |  |
|  |  |  |  |  |  |  |  |  |  |  |  |  |  |  |  |  |  |  |  |  |
|  | Gp10 |  |  |  |  |  |  |  |  |  |  |  |  |  |  |  |  |  | -0.469 |  |
|  |  |  |  |  |  |  |  |  |  |  |  |  |  |  |  |  |  |  | **0.048** |  |
|  | Gp11 |  |  |  |  |  |  |  |  |  |  |  |  |  |  |  |  |  |  |  |
|  |  |  |  |  |  |  |  |  |  |  |  |  |  |  |  |  |  |  |  |  |
|  | Gp13 |  |  |  |  |  |  |  |  |  |  |  |  |  |  |  |  |  |  |  |
|  |  |  |  |  |  |  |  |  |  |  |  |  |  |  |  |  |  |  |  |  |
|  | Gp17 |  |  |  |  |  |  | -0.639 |  |  |  |  |  |  |  |  |  |  |  |  |
|  |  |  |  |  |  |  |  | **0.004** |  |  |  |  |  |  |  |  |  |  |  |  |
|  | Gp18 |  |  |  |  |  |  |  |  |  |  |  |  |  |  |  |  |  |  |  |
|  |  |  |  |  |  |  |  |  |  |  |  |  |  |  |  |  |  |  |  |  |
|  | Gp21 |  |  |  |  |  |  |  |  |  |  |  |  |  |  |  | 0.625 |  |  |  |
|  |  |  |  |  |  |  |  |  |  |  |  |  |  |  |  |  | **0.005** |  |  |  |
|  | Gp22 |  |  |  |  |  |  |  |  |  |  |  |  |  |  |  |  |  |  |  |
|  |  |  |  |  |  |  |  |  |  |  |  |  |  |  |  |  |  |  |  |  |
|  | Gp23 |  |  |  |  |  |  |  |  |  |  |  |  |  |  |  |  |  |  |  |
|  |  |  |  |  |  |  |  |  |  |  |  |  |  |  |  |  |  |  |  |  |
|  | Gp25 |  |  |  |  |  |  |  |  |  |  |  |  |  |  |  |  |  |  |  |
|  |  |  |  |  |  |  |  |  |  |  |  |  |  |  |  |  |  |  |  |  |
|  | Gp26 |  |  |  |  |  |  |  |  |  |  |  |  |  |  |  |  |  |  |  |
|  | Others |  |  |  | -0.5 |  | 0.707 |  |  |  |  |  | 0.562 |  |  |  |  |  |  | 1 |
|  |  |  |  |  | **0.04** |  | **0.0008** |  |  |  |  |  | **0.015** |  |  |  |  |  |  | **0.0000002** |

*Spearman* ´s correlation coefficient; Highlighted: value of significance *P* ≤ 0.05
